# Multi-step proportional miniaturization to sub-micron dimensions using pre-stressed polymer films

**DOI:** 10.1039/d0na00785d

**Published:** 2020-10-26

**Authors:** Shady Sayed, P. Ravi Selvaganapathy

**Affiliations:** Department of Mechanical Engineering, McMaster University Hamilton ON L8S 4L8 Canada selvaga@mcmaster.ca

## Abstract

The ability to define patterns and fabricate structures at the nanoscale in a scalable manner is crucial not only in integrated circuit fabrication but also in fabrication of nanofluidic devices as well as in nano and micromechanical systems. Top down fabrication at the nanoscale often involves fabrication of a master using a direct write method and then its replication using a variety of methods such as by hot embossing, nanoimprint lithography, or soft lithography. Nevertheless, fabrication of the master is a time consuming and expensive process. One interesting approach is to define patterns at larger dimensions on pre-stressed films using methods such as xurography or lithography which are scalable and heat them to de-stress and shrink which can reduce the size proportionally. Although attractive, suitable fabrication processes that can perform iterative shrinking of patterns over several cycles and into the nanoscale have not been demonstrated. Here, we demonstrate a fabrication process that is capable of accurately producing patterns and features over several cycles of miniaturization and shrinking to achieve resolution in the order of 100 s of nanometers. In this approach, a pattern transfer method is developed by combining soft imprint lithography followed by reactive ion etching, both of which are scalable processes, to transfer the original patterns into a shrinkable polymer film. The patterned shrinkable film is heated to allow thermal shrinking. As a result, the pattern size was decreased by 60% of the original size in a single cycle. This reduced pattern was used as the master for the next cycle and three cycles of miniaturization was demonstrated. Sub-micron patterns of 750 nm were generated by the multi-step miniaturization method, showing approximately 20× reduction in size of the original patterns. Finally, these patterns are transferred into features on a silicon substrate to demonstrate its application in semiconductor microfabrication or its use as a master template for microsystems applications.

## Abstract 2

Multi-step miniaturization to sub-micron dimensions using shrinkable polymer films.

## Graphical abstract

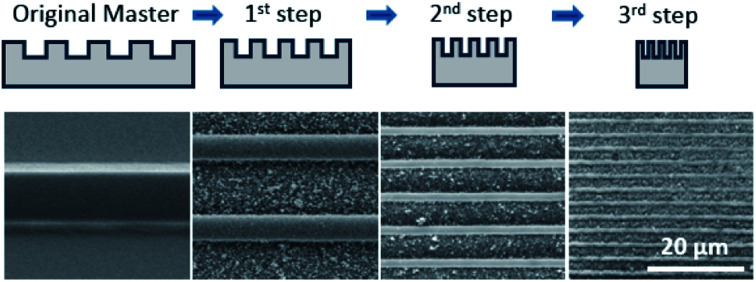

## I. Introduction

Fabrication of patterns and features in the nanoscale is important in many applications such as microelectronics, photonics, nanosystems and nanofluidics. Methods such as photolithography,^1^ nano imprint lithography^2^ and soft lithography^3^ exist for production of such patterns but require an expensively produced mask^4^ or master mold which are often made using direct write approaches such as electron beam lithography or focused ion beam lithography^5^ that consume a significant amount of time to produce complex and intricate features. An alternate process that can simplify production of mask patterns or master molds in a low cost, scalable manner can be effective in reducing the cost and complexity associated with nanofabrication. One approach that has been investigated is to define patterns in the scale of hundreds of micrometers on pre-stressed, mechanically stretched polymeric films and to proportionally reduce their feature sizes by using a thermal trigger that relaxes the internal stresses and shrinks the film to dimensions that are a few tens of micrometers.^6–8^ When these stretched polymer films are heated slightly above the glass transition temperature, the polymer reflows just enough to release the compressive stresses embedded in it which tends to recover to its original shape and up to 77% reduction in size is possible. When the shrinkable films are patterned, these patterns also tend to shrink in dimension when the entire film is exposed to the heat trigger and relaxes. Many different methods including reactive ion etching (RIE),^9^ hot embossing,^10^ and contact etching lithography^11^ have been used to embed the pattern directly on to the heat shrink film itself. Although these methods have been used to miniaturize patterns, they are unsuitable for repeated operation where the shrunk pattern itself is used as a master for the next cycle to enable their continued miniaturization into sub micrometer dimensions. For instance, the use of hot embossing and contact lithography produce shrunk patterns that have much lower aspect ratio than the original master which leads to loss of resolution in the next cycle. RIE patterns typically are generated using hard metal masks that are difficult to produce from the shrunk patterns generated from them for continued miniaturization.

Alternatively, soft lithography,^12^ and photolithography^13^ have been used to pattern other materials on top of the heat shrink films which can enable miniaturization of such patterns. The limitation of this method is that the presence of another material can restrict the miniaturization of that region while the space between the patterns are miniaturized. Therefore, proportional miniaturization of the feature and the spacing between them is not possible.

In general, existing methods are suited for a single step miniaturization where the existing pattern is reduced by 30–70% of its original size. However, they are not ideally suited for a multi cycle miniaturization process where the shrunk pattern from the previous cycle can be used as a master for the next cycle for scalable miniaturization to sub micrometer and nanometer dimensions.

Here, we report a scalable miniaturization approach using heat shrinkable Polystyrene (PS) films that generates a proportionally miniaturized pattern which can in-turn be used as master for the next cycle of miniaturization. We demonstrate that this approach can be used multiple times to fabricate sub-micron patterns. Proportional reduction in dimensional features of 20 times were demonstrated over three cycles of miniaturization. Starting from 15 μm features of the master, patterns as small as 750 nm were fabricated. We applied this new capability to fabricate patterns in a silicon wafer that can be used as a functional substrate for many applications such as diffraction grating based sensors^14,15^ or as a master template for soft lithography applications.^16,17^

## II. Experimental

**Fig. 1:**
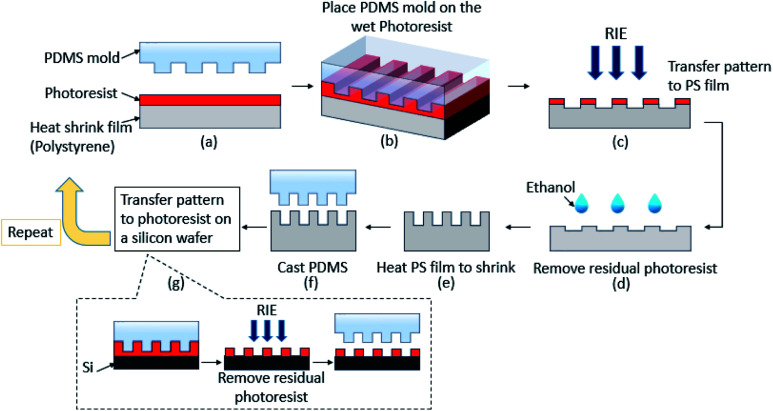
Schematic illustration of the multi-step miniaturization process using heat shrinkable films. (a)–(g) Sequential steps of the fabrication process.

A multi-step miniaturization approach was developed that can be used repeatedly to produce patterns that are progressively smaller than the patterns in the previous step for scalable and continued miniaturization. A schematic illustration of the multi-step miniaturization process is shown in Fig. 1. First, the original master pattern substrate was fabricated lithographically by patterning a photoresist (Microposit S1827) coated on a silicon wafer. Although lithography was used here, the master can be fabricated by other methods such as laser machining or xurography as well. Two different patterns were fabricated, namely: line-space pattern with line width of 15 μm and pillars pattern of 15 μm diameter. These dimensions are easy to produce using a variety of microscale patterning methods and hence were chosen. Then, an elastomer, poly(dimethylsiloxane) (PDMS, Sylgard 184, Dow Corning), was molded on the fabricated master by replica molding to be used as a master template. A photoresist (AZ-MIR 701) was spin-coated on the heat shrinkable polystyrene film (Grafix) at 2000 rpm for 15 seconds, which ensured that the photoresist was uniformly distributed but still wet (Fig. 1a). By soft imprint lithography, the PDMS mold was placed immediately on the coated photoresist to stamp it and transfer the pattern on to the photoresist layer (Fig. 1b). The patterned photoresist was used as a mask in the subsequent RIE process (Fig. 1c). The PDMS mold was held in place for 10 min at room temperature to allow the photoresist to dry and then carefully removed. After patterning the photoresist, the sample was exposed to an O_2_ plasma in a RIE chamber (Technics) (power 100 W, flow rate 16 sccm, pressure 210 mTorr) for 18–25 min. Both the photoresist mask and the heat shrinkable film were etched which leads to transfer the pattern into the shrinkable film itself.

The remaining photoresist was removed by a quick rinse with ethanol followed by deionized water (Fig. 1d). The PS film was heated in an oven at 135 °C for 15 min which allows it to thermally shrink by approximately 60% of the original size (Fig. 1e). PDMS was cast onto the shrunk pattern to form a mold that was used as a new template for further miniaturization steps (Fig. 1f). To improve the surface finish of the generated pattern which has a significant effect on the fidelity of the subsequent miniaturization steps, a silicon wafer coated with a photoresist was patterned by same soft imprint lithography process using the PDMS mold formed from the shrunk pattern (Fig. 1g). Then the residual photoresist layer was etched by O_2_ plasma RIE leaving behind clean and smooth Si surface in the regions where the photoresist pattern was not present. PDMS was then cast on this pattern to form the PDMS mold which was used for the next miniaturization step.

The combination of soft imprint lithography, reactive ion etching of heat shrinkable films as well as the use of the intermediate Si-photoresist hybrid mold, makes it possible to use the shrunk patterns of the previous step as a new master for the next step in a multi-step miniaturization that can be repeated multiple times to obtain nanoscale resolution. Thus, from a single master pattern, different smaller size patterns can be fabricated.

## III. Results and discussion

### Multi-step miniaturization process

**Fig. 2:**
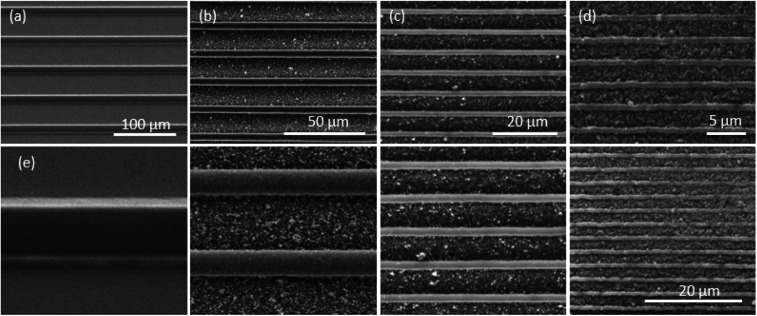
SEM images of the line-space pattern (a) master pattern (line-width *w* = 15 μm, spacing *s* = 30 μm), (b) first step miniaturization (*w* = 5.4 μm, *s* = 11 μm), (c) second step (*w* = 2 μm, *s* = 4.5 μm), (d) third step (*w* = 0.75 μm, *s* = 1.9 μm), (e) the master and three miniaturization steps at same magnification.

Pre-stressed polymer films have been used to demonstrate single step miniaturization of patterns. Previous attempts at multi step miniaturization have led to loss of resolution and fidelity after the 1^st^ or the 2^nd^ steps.^7,11^ Therefore, in order to demonstrate the scalability of the newly established process (Fig. 1), a three step miniaturization sequence was used to show that large line patterns can be reduced to sub micrometer dimension. The initial master (Fig. 2a) was fabricated lithographically as a line pattern with 15 μm line width and 30 μm spacing. These dimensions were chosen as they are easily achievable by low cost methods such as laser writing or 3D printing using stereolithography methods. This pattern was successively miniaturized three times with the final shrunk pattern of the previous step serving as the master mold for the next step to reduce the final dimension to 750 nm line width which is a 20× reduction in size of the original pattern. SEM images were taken at each step which are shown in Fig. 2 (a–e). After the first miniaturization step (Fig. 2b) the line width reduces to 5.4 μm (64% reduction) and the spacing to 11 μm. After the second step (Fig. 2c), the line width reduces further to 2 μm (63% reduction) and the spacing to 4.5 μm. Finally, after the third step (Fig. 2d), the line width reduces to 750 nm (62.5% reduction) and the spacing to 1.9 μm. It is interesting to note that the fidelity of the pattern is retained even when its size is reduced by ∼20× to sub micrometer dimensions. Also, the original ratio of 0.5 (line width to spacing) was found to be maintained after the first step at 0.49 while it reduced to 0.44 and 0.4 in the subsequent steps. These results align with the expected behavior of these pre-stressed films which shrink by 60–65% in both the lateral directions. The design of the fabrication process such that the shrunk pattern of one step can be used as the master of the next can be used to progressively achieve smaller and smaller patterns without any expensive equipment. The fidelity of the pattern has been shown to be maintained throughout the successive miniaturization step.

**Fig. 3:**
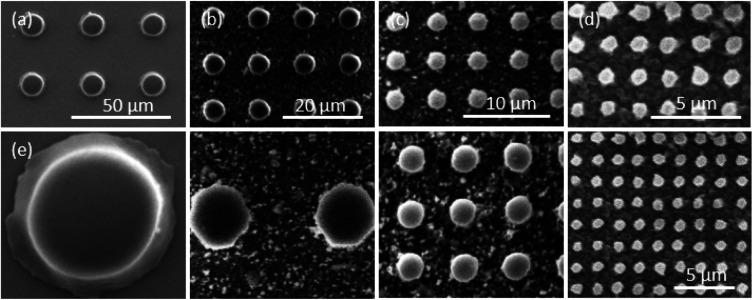
SEM images of the pillars pattern (a) master pattern (diameter *d* = 14.5 μm, spacing *s* = 15.5 μm), (b) first step miniaturization (*d* = 5.5 μm, *s* = 5.8 μm), (c) second step (*d* = 2.2 μm, *s* = 2.3 μm), (d) third step (*d* = 0.86 μm, *s* = 0.9 μm), (e) the master and three miniaturization steps at same magnification.

In order to show that the fabrication process can also be used to create features other than line patterns, a pillar array pattern was also microfabricated using the multi-step miniaturization process. The initial master (Fig. 3a) was a pillar pattern with 14.5 μm diameter and 15.5 μm spacing which was fabricated lithographically. Using the same multi-step miniaturization process, this was reduced in size over three steps. SEM images were taken at each step which are shown in Fig. 3 (a–e). After the first miniaturization step (Fig. 3b) the diameter reduced to 5.5 μm (62% reduction) and the spacing to 5.8 μm. After the second step (Fig. 3c), the diameter reduced further to 2.2 μm (60% reduction) and the spacing to 2.3 μm. Finally, after the third step (Fig. 3d), the diameter reduced to 860 nm (61% reduction) and the spacing to 900 nm. The fidelity of the pattern is retained over the three miniaturization cycles. In addition, the original ratio of 0.935 (diameter to spacing) was found to be slightly increased after the first step to 0.95 while it maintained constant at 0.955 in the subsequent steps. The results show that smaller size patterns can be generated from a single master and can be applied to different shape features.

### Eliminating surface roughness accumulation in each step

One of the challenges in a multi-step miniaturization process is that defects that originate in earlier steps accumulate in subsequent steps and leads quickly to loss of fidelity. This effect is particularly important in miniaturization of patterns using pre-stressed films and has led to previous attempts being restricted to only two steps or to large feature sizes.^11,13^ In the current fabrication process, the pre-stressed PS film is etched by RIE which leaves behind a rough surface in the exposed and etched regions. During the shrinking process the lateral dimensions of the film reduces by 60–65% while the vertical dimension of the features in it increase by a factor of ∼6.25 due to volume conservation. This increase in height is desired to amplify the etched patterns embedded into these films; however, they also amplify the surface roughness in the etched regions. Surface roughness can be detrimental as they create stress concentrations and can cause distorted shrinking of the films in subsequent steps. This effect becomes particularly important when the dimensions of the patterns are close to 1 μm or below. The use of a Si wafer intermediate in the process to form the mold for the next step can potentially mitigate the surface roughness in the etched regions due to RIE.

**Fig. 4:**
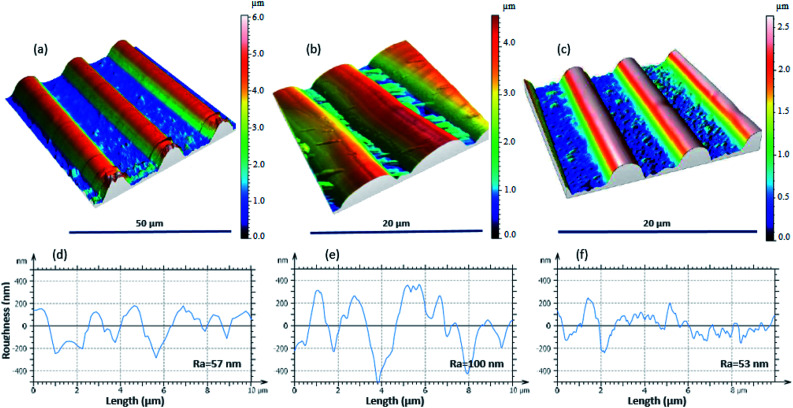
AFM images and the corresponding surface roughness measurements of (a and d) the first miniaturization step, (b and e) second miniaturization step directly, (c and f) second miniaturization step after modification by using Si wafer.

In order to demonstrate the effect of the intermediate step, experiments were performed wherein the PDMS replica mold of the shrunk pattern was used to pattern a photoresist coated on a Si wafer by soft imprint lithography (Fig. 1g). Then, the residual photoresist layer (which produced by the undesired rough surface of the shrunk pattern) was removed by a short O_2_ RIE. A new PDMS mold was then cast on the patterned Si wafer and used to pattern the next miniaturization step. The PDMS mold directly made from the shrunk pattern was used as a control. Atomic force microscopy was performed (Fig. 4) on the patterns comparing the surface topography after the first miniaturization step (Fig. 4a) and those formed after the second miniaturization step to determine the effect of the use of the Si intermediate mold (Fig. 4c) *vs.* the control case (Fig. 4b). It shows clearly that the surface roughness of the etched regions was significantly lower when the Si intermediate mold was used to then subsequently transfer the shrunk pattern to PDMS. The average surface roughness of the first miniaturization step after shrinkage was found to be ∼57 nm, (Fig. 4d). The surface roughness of the patterns generated after the second step of miniaturization was only ∼53 nm (essentially unchanged, Fig. 4f) when the Si intermediate mold was used while it increased to ∼100 nm (Fig. 4e) in the control case where the PDMS mold was created directly from the shrunk pattern of the previous step. These results show that the use of Si intermediate mold was able to mitigate the increase in surface roughness that occurs when the imprinting mold for the next step is directly cast from the shrunk mold of the previous step. This increase in surface roughness in the control case results in loss of resolution in each successive step and prevents the use of this method beyond one or two successive step. With the Si intermediate mold, successive miniaturization can be carried out indefinitely until the dimensions of the patterns approach the surface roughness of the heat shrinking polymer film.

### Pattern transfer from heat shrunk polymer films onto functional substrates

**Fig. 5:**
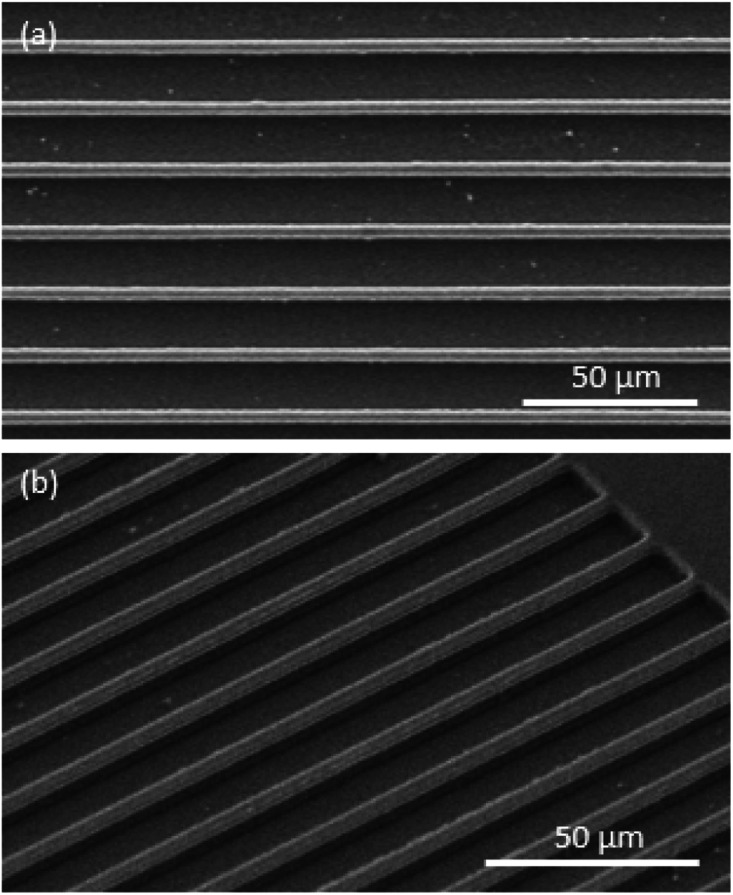
SEM images of the fabricated Si wafer after transferring the pattern of the shrunk film (a) top view, (b) incline view (tilt angle of 45°).

The multi-step miniaturization process is used to reduce the dimensions of the patterns after which the reduced patterns have to be transferred on to a functional substrate. In order to demonstrate the pattern transfer and the features that are produced, Si was chosen as a functional substrate as it is widely used in electronic applications. The PDMS mold of the shrunk pattern after the first step of miniaturization (Fig. 2b) was used to pattern a photoresist coated on a Si substrate by soft imprint lithography. Then the patterned substrate was exposed to a short O_2_ RIE (20 s) to remove any residual photoresist layer. The patterned photoresist was used as a mask to etch the Si substrate by RIE with mixed gases (C_4_F_8_ and SF_6_) for 4–6 min. The remaining photoresist mask was then removed by acetone or by O_2_ plasma RIE. Fig. 5 shows SEM images of the fabricated Si substrate after transferring the pattern. A top view of the patterned Si substrate is shown in Fig. 5a, and an incline view is shown in Fig. 5b. The line width is 5.2 μm, the spacing is 11.6 μm and the depth of the etched pattern is 4.8 μm. It clearly illustrates that the shrunk patterns generated from the miniaturization process can be transferred into a Si substrate in large-area and deep patterns.

### Effect of aspect ratio of patterns on miniaturization

**Fig. 6:**
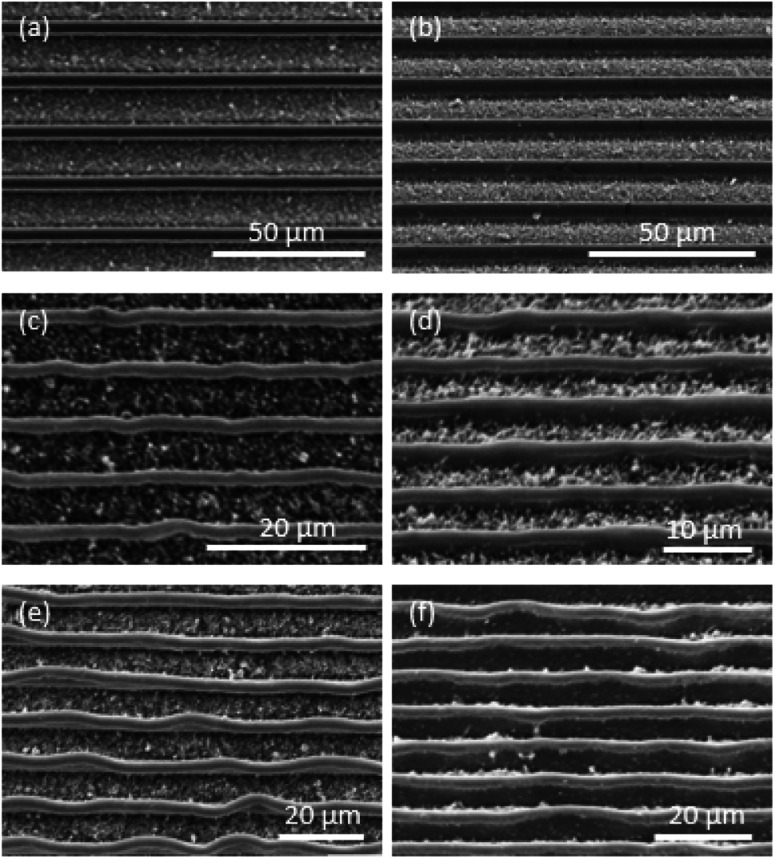
SEM images of the line-space pattern at different aspect ratios (*λ*). The aspect ratios are (a and b) *λ* = 1, (c and d) *λ* = 1.5, and (e and f) *λ* = 2. (a, c and e) are top views and (b, d and f) are incline views.

The height of the patterns formed after the RIE of the pre-stressed polymer films has a significant effect on the fidelity of the features formed after heat induced shrinking. This effect will then have a significant influence on the ability to miniaturize over a number of steps. In order to identify the appropriate aspect ratio of the etched features that would be suitable, line-space patterns were fabricated at different aspect ratios (*λ*) which is defined as the ratio between the height of the pattern after shrinkage and the line width. The height of the features was controlled by the depth of etch during the RIE process. The SEM images of the patterns formed after shrinking are shown in both the top down (Fig. 6a, c and e) and inclined (Fig. 6b, d and f). When the aspect ratio was *λ* = 1 (Fig. 6a and b), the shrunk pattern had good fidelity with the original etched pattern and the lines were straight. At *λ* = 1.5 (Fig. 6c and d), the shrunk pattern began to lose some fidelity and was found to contain some kinks that were not present in the original etched pattern. These deformations were found to increase with the aspect ratio. For instance, when *λ* = 2, large deformations were seen (Fig. 6e and f). Thus *λ* = 1 is suitable for proportional miniaturization of patterns in this format. This finding is important as the aspect ratio of the features etched into the pre-stressed polymer film has to be maintained at one in order to get the best feature fidelity. It implies that the etch depth of the features embedded into the polymer film such be reduced in each successive step of miniaturization. However, an etch depth that is comparable to the surface roughness of the pre-stressed film will also lead to the loss of fidelity. Therefore, the surface roughness of the pre-stressed film determines the minimum feature size that is achievable through this successive miniaturization process.

## IV. Conclusions

In summary, we have demonstrated a simple yet powerful multi-step miniaturization approach using pre-stressed polymer films that offers a sequential size reduction of micron and sub-micron patterns. A pattern transfer method was developed by a combination of soft imprint lithography and RIE that offers a way to use the shrunk patterns as new masters without the need of fabricating hard or metal templates. Thus, from a single master, new masters are created with different smaller feature sizes. A sequential miniaturization of different patterns have been demonstrated showing a 20× reduction in size of the original patterns, and achieving features as small as 750 nm. Moreover, to show that the fabricated patterns are not limited to polymeric materials and can be converted into a functional substrate, they are transferred to a Si substrate. These capabilities are promising in micro and nano fabrication, and offer significant advantages over conventional photolithography in terms of resolution and over advanced lithography methods such as nanoimprint lithography in terms of cost. In addition, the multi-step miniaturization approach introduces a new micron/sub-micron fabrication method that can be performed in any lab without the need of expensive instruments.

## Conflicts of interest

There are no conflicts to declare.

## Supplementary Material
